# When Transfer Is Impossible: Emergency Management of Perforated Necrotizing Enterocolitis in a Neonate With Uncorrected Dextro-Transposition of the Great Arteries

**DOI:** 10.7759/cureus.114544

**Published:** 2026-08-14

**Authors:** Verónica Eckardt, Daniel Gao, Céline Marques

**Affiliations:** 1 Anesthesiology, Unidade Local de Saúde de Lisboa Ocidental, Lisbon, PRT; 2 Anesthesiology, Unidade Local de Saúde de São José, Lisbon, PRT; 3 Anesthesiology, Hospital dos Serviços de Assistência Médico-Social, Lisbon, PRT

**Keywords:** cardiogenic necrotizing enterocolitis, dextro-transposition of the great arteries, left ventricular outflow tract obstruction (lvot), near-infrared spectroscopy (nirs), necrotizing enterocolitis (nec), neonatal anesthesia, neonatal emergency management, pediatric cardiac anesthesia, pediatric surgery emergency management, ventricular septal defect (vsd)

## Abstract

Dextro-transposition of the great arteries (d-TGA) is a congenital heart disease (CHD) characterized by parallel circulations, in which survival depends on intercirculatory mixing. CHD as a whole is a recognized risk factor for necrotizing enterocolitis (NEC), which carries high morbidity and mortality. Managing perforated NEC in a neonate with uncorrected complex d-TGA presents a complex challenge, particularly in centers lacking specialized pediatric cardiac surgery facilities.

We report the successful emergency management of a 28-day-old preterm neonate with uncorrected complex d-TGA, a large ventricular septal defect, and severe subpulmonary obstruction requiring urgent surgery. Due to vital urgency and the impossibility of transfer to a specialized center with pediatric cardiac surgery, local care was optimized through remote expert consultation and an intensive multidisciplinary briefing. Anesthesia was tailored to maintain systemic vascular resistance and preserve pressure-driven intercirculatory mixing. The team relied on multimodal noninvasive monitoring, integrating cerebral near-infrared spectroscopy (NIRS) and continuous noninvasive hemoglobin (SpHb). Cerebral NIRS complemented conventional monitoring, detecting a drop in regional oxygen saturation (rSO2) and prompting a proactive transfusion strategy that stabilized the infant intraoperatively. Following a successful left hemicolectomy, the infant was later transferred to a specialized cardiac center for further management of d-TGA.

Although surgery in such critical neonates is ideally performed in specialized centers, this case demonstrates how leveraging multidisciplinary teamwork, advanced monitoring, and physiological understanding can bridge the gap between gold standards and the practical realities of life-threatening neonatal emergencies in nonspecialized facilities.

## Introduction

Dextro-transposition of the great arteries (d-TGA) accounts for 5-7% of all congenital heart diseases (CHD) and is characterized by ventriculoarterial discordance: the normal atrioventricular connections are preserved, but the aorta arises from the right ventricle (RV) and the pulmonary artery from the left ventricle (LV) [1]. As a result, systemic and pulmonary circulations run in parallel, which makes survival depend on obligatory mixing between the two circuits [1]. Common mixing sites include patent foramen ovale, patent ductus arteriosus (PDA), and ventricular septal defects (VSDs), with VSDs defining complex d-TGA [1]. In the present case, the large VSD was the dominant mixing site, and severe subpulmonary obstruction raised pressure within the LV (subpulmonary ventricle), augmenting a left-to-right shunt across the VSD that diverted oxygenated blood into the RV (subaortic ventricle) and aorta, supporting systemic oxygen delivery.

In parallel, necrotizing enterocolitis (NEC) is a severe disease of the gastrointestinal tract characterized by inflammation, ischemia, and necrosis of the bowel wall, with reported mortality rates between 10% and 50% in affected infants [2,3]. CHD is a well-recognized risk factor in both term and preterm infants, with an incidence of 1.6-6% in full-term neonates with CHD [2,3]. There is growing recognition that cardiogenic NEC (cNEC) is a distinct clinical entity rather than a variant of classical NEC of prematurity: while prematurity-associated NEC typically affects the small bowel, cNEC in term or late-preterm infants with CHD more often involves the colon and is thought to derive from low systemic output, impaired mesenteric perfusion, abnormal diastolic flow reversal, chronic hypoxemia, and inflammatory stress rather than mucosal immaturity alone [4,5]. This distinction complicates diagnosis relative to classic prematurity-associated NEC, since abdominal deterioration may reflect both primary bowel pathology and circulatory compromise [3,5]. A low threshold for imaging and surgical assessment is always warranted [4,5]. When CHD is complicated by perforated NEC, these two overlapping pathophysiologies converge into a single critical clinical challenge, in which resuscitation goals for one condition may worsen the other by reducing the pressure gradient that drives both systemic perfusion and intercirculatory mixing.

Currently, there is limited published evidence regarding the anesthetic management of patients with uncorrected d-TGA undergoing noncardiac surgery [6]. We report the successful management of a 28-day-old preterm neonate with d-TGA, a large VSD, and severe subpulmonary obstruction who developed perforated NEC and had to undergo an emergency laparotomy. The procedure was performed in a pediatric center without cardiac surgery, where preoperative planning and multimodal monitoring were essential to overcome the impossibility of interhospital transfer.

## Case presentation

A preterm male neonate was prenatally diagnosed with d-TGA at 26 weeks' gestation, subsequently confirmed by serial fetal echocardiography, which identified a large perimembranous VSD and mild subpulmonary stenosis. He was born at 34 weeks and four days of gestation by cesarean section for worsening maternal pre-eclampsia. At 28 days of life, he developed late-onset sepsis and perforated NEC with diffuse peritonitis.

The team faced a difficult decision between delaying surgery for transfer to a specialized center and proceeding locally. Given the patient's rapidly deteriorating condition and vital urgency, transfer to a pediatric cardiac center was not possible. The case was therefore managed at a nonspecialized pediatric center, and the patient underwent an emergency exploratory laparotomy. A pediatric cardiac anesthesiologist was consulted remotely. A multidisciplinary briefing involving surgeons, anesthesiologists, intensivists, and nurses defined the operative steps, including preparation of vasoactive agent infusions and review of the cardiopulmonary resuscitation protocol in case of need.

**Figure 1:**
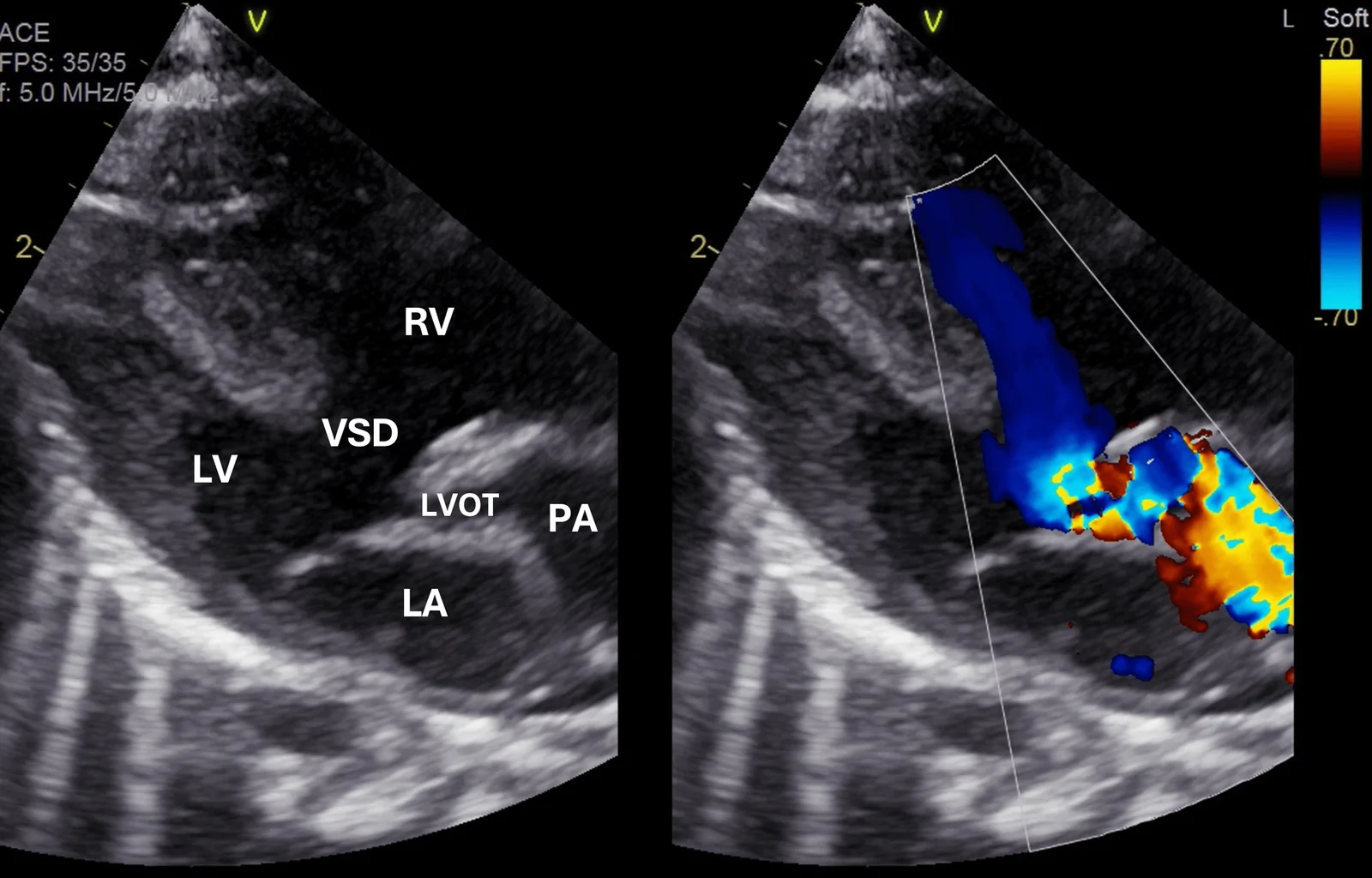
Preoperative echocardiogram. (Left) Two-dimensional image showing the right ventricle, left ventricle, left atrium, pulmonary artery, and the perimembranous ventricular septal defect. (Right) Corresponding color-flow Doppler image demonstrating turbulent flow across the ventricular septal defect and the left ventricular outflow tract. LA, left atrium; LV, left ventricle; LVOT, left ventricular outflow tract; PA, pulmonary artery; RV, right ventricle; VSD, ventricular septal defect.

**Figure 2:**
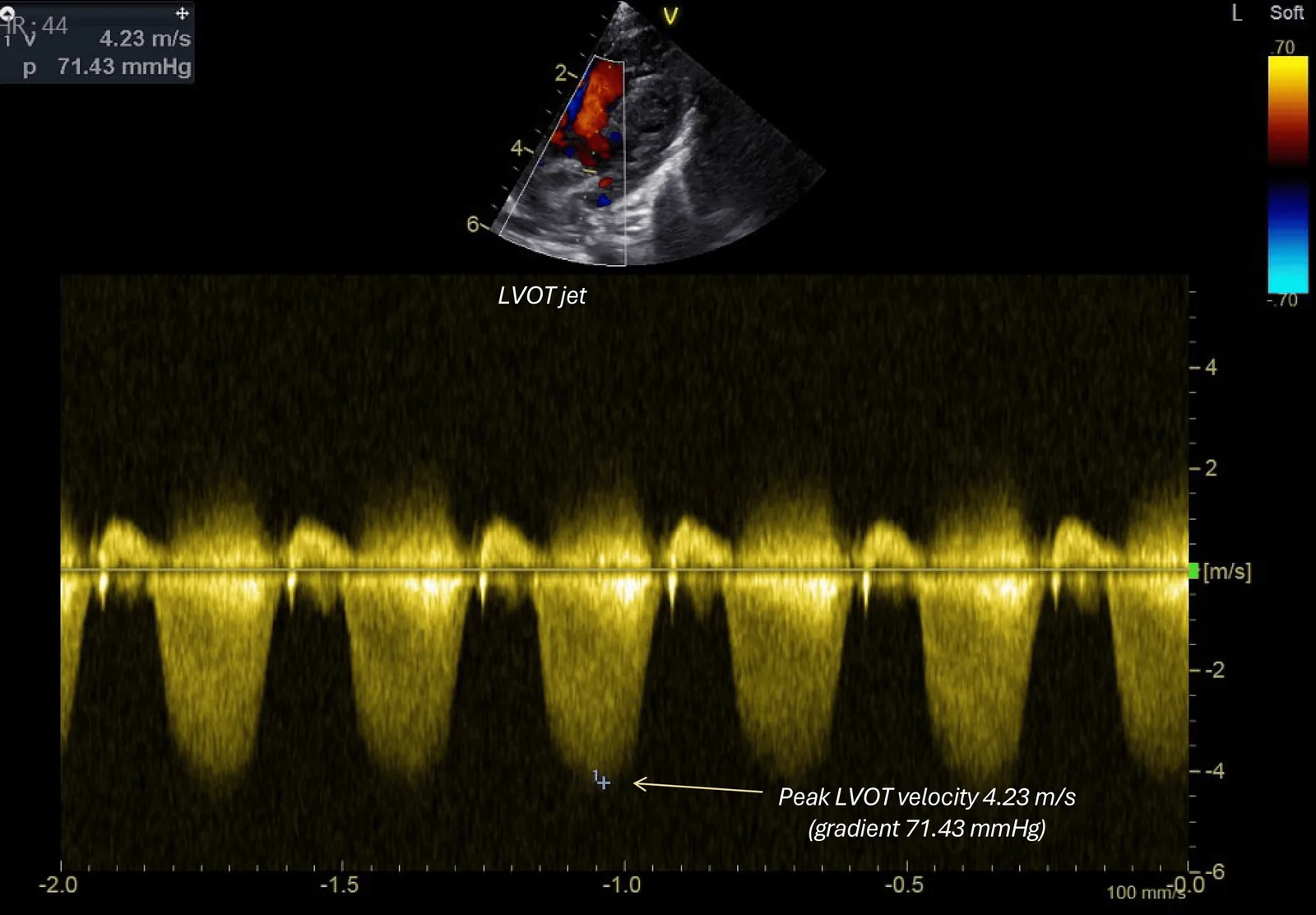
Preoperative continuous-wave Doppler across the left ventricular outflow tract. Spectral trace demonstrating a peak velocity of 4.23 m/s, corresponding to a peak instantaneous pressure gradient of 71.43 mmHg. LVOT, left ventricular outflow tract.

Preoperatively, the 28-day-old neonate, weighing 2.6 kg, was in critical condition, presenting with hypothermia (35.3°C), poor activity, and pale, mottled skin, with a heart rate (HR) of 147 bpm and a mean arterial pressure (MAP) of 54-57 mmHg. He had already been intubated on day 27 and was maintained on invasive mechanical ventilation. Despite receiving an FiO2 of 40-70%, his peripheral oxygen saturation (SpO2) remained at 80-81%. Physical examination revealed a distended and tender abdomen. Echocardiography showed d-TGA with a large perimembranous VSD (Figure 1) and significant subpulmonary obstruction (5 mm; Z-score -1.84), characterized by posterior deviation of the infundibular septum and marked narrowing of the LVOT (1.5-2 mm), resulting in a peak pressure gradient of 45-72 mmHg (Figure 2). 

A small patent ductus arteriosus (PDA) and a small aortopulmonary collateral were present, and he showed good biventricular function. Relevant preoperative laboratory values, alongside neonatal reference ranges, are summarized in Table 1. The markedly elevated C-reactive protein supported the diagnosis of NEC-associated sepsis over isolated cardiac deterioration.

**Table 1:** Pre- and postoperative laboratory values. APTT, activated partial thromboplastin time; Ca, calcium; Cl⁻, chloride; CRP, C-reactive protein; Hb, hemoglobin; Hct, hematocrit; INR, international normalized ratio; K⁺, potassium; Mg, magnesium; Na⁺, sodium; P, phosphate; PT, prothrombin time; WBC, white blood cells.

Parameter	Pre-operative	Post-operative	Neonatal reference range	Units
Hemoglobin (Hb)	14.1	11.2	13.5–18.5	g/dL
Hematocrit (Hct)	41.2	31.2	42–60	%
White blood cells (WBC)	14,650	19,190	5,000–19,500	/µL
Platelets	91,000	84,000	150,000–450,000	/µL
Lactate	0.3	0.6	0.5–2.2	mmol/L
C-reactive protein (CRP)	135.9	109.7	<10	mg/L
Prothrombin time (PT)	18.5	—	9.5–12.6	sec
International normalized ratio (INR)	1.52	—	1.1–1.7	—
Activated partial thromboplastin time (APTT)	50.2 (ratio 1.7)	—	28–46	sec
Fibrinogen	2.6	—	1.4–3.0	g/L
Urea	13	16	5–20	mg/dL
Creatinine	0.16	0.15	0.2–0.4	mg/dL
Sodium (Na+)	136	136	135–145	mEq/L
Potassium (K+)	4.8	4.8	3.5–5.5	mEq/L
Chloride (Cl-)	103	103	95–110	mEq/L
Total calcium (Ca)	9.2	7.8	8.5–10.5	mg/dL
Phosphate (P)	5.2	5.6	4.5–8.0	mg/dL
Magnesium (Mg)	1.94	—	1.5–2.5	mg/dL

Preoperative optimization focused on maintaining invasive mechanical ventilation, active warming to reach a temperature of 36.5°C, and systemic analgesia with continuous fentanyl. Further stabilization included maintaining a peripheral infusion of balanced electrolyte solutions at a rate of approximately 5 mL/kg/h and reserving blood components.

Intraoperatively, balanced general anesthesia was maintained with fentanyl (titrated to 1.4-2.0 mcg/kg/h), sevoflurane (minimum alveolar concentration 0.6-1), ketamine (5 mg total), and rocuronium (6.5 mg total). Monitoring included five-lead electrocardiography, venous blood gas analysis, serum glucose, serum potassium, serum calcium, urine output, cerebral oximetry (rSO2, Medtronic, Minneapolis, Minnesota), and continuous hemoglobin (SpHb, Masimo, Irvine, California), with a basal SpO2 of 81%, rSO2 of 75% at 85% FiO2, and SpHb of 14 g/dL. Normothermia was maintained using forced-air warming, a heating mattress, and warmed fluids. A nasogastric tube with active low-pressure suction was in place. Two peripheral intravenous catheters and an ultrasound-guided brachiocephalic vein central venous catheter were placed. Initial attempts by the neonatology intensivist team to establish an arterial line were unsuccessful due to severe peripheral vasoconstriction and technical challenges inherent to prematurity. Because repeated arterial cannulation attempts were considered unlikely to succeed without delaying surgery, management proceeded using multimodal noninvasive monitoring. To mitigate the high risk of cardiac arrest, external defibrillation pads were placed prior to the induction of anesthesia.

**Figure 3:**
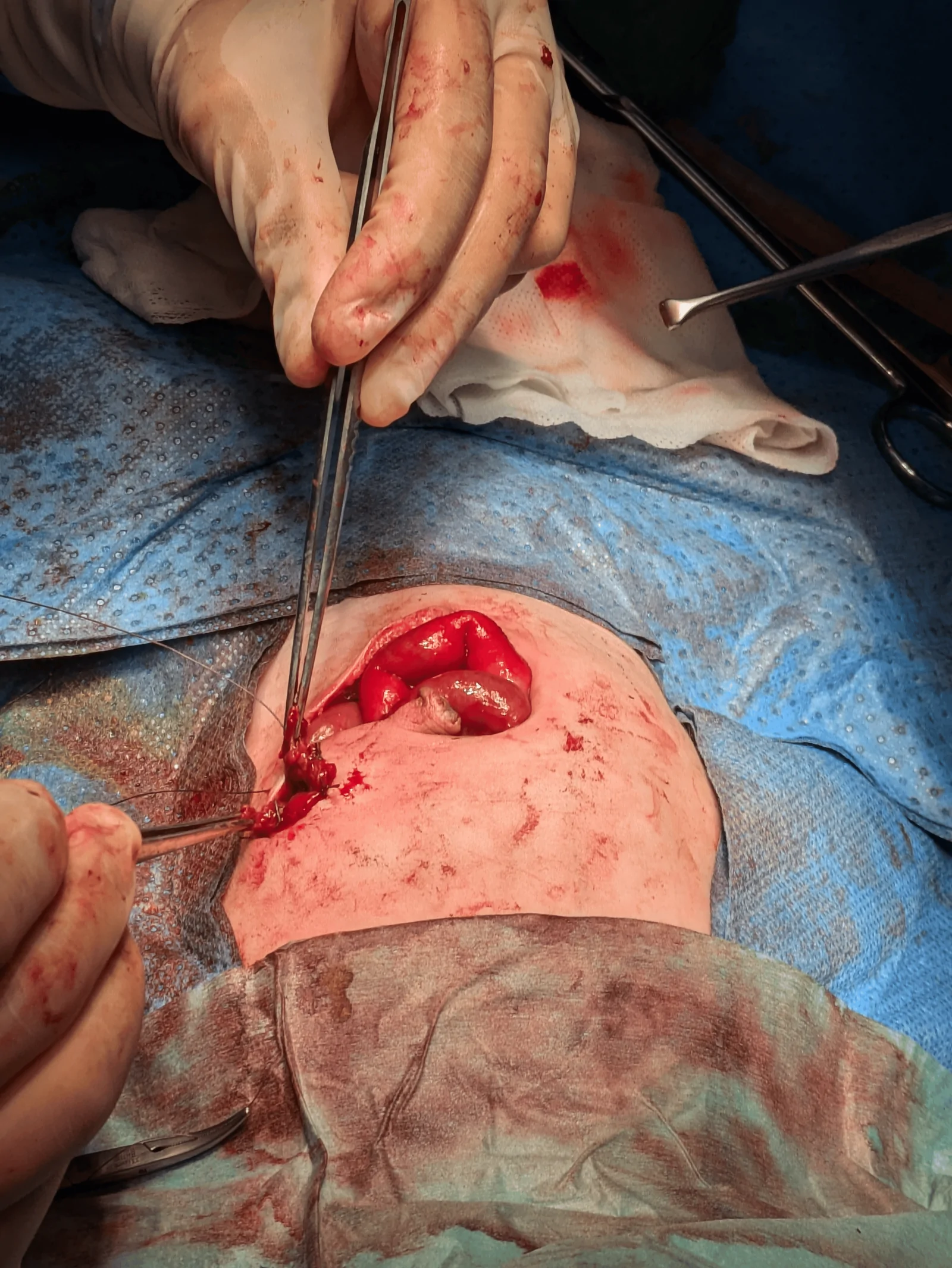
Intraoperative photograph of left hemicolectomy with transversostomy.

During the laparotomy, despite initial fluid resuscitation with a 10 mL/kg crystalloid bolus, cerebral rSO2 fell from a baseline of 75% (at FiO2 85%) to 58%, together with a decrease in EtCO2. The team decided to transfuse red blood cells (10 mL/kg) in response. Over the following minutes, EtCO2 fell to 22 mmHg, MAP fell to 40 mmHg, and heart rate rose to 189 bpm, while peripheral SpO2 remained comparatively preserved (85-89%) and SpHb fell to 10 g/dL. A second red blood cell bolus of the same volume was then administered (total 20 mL/kg), after which rSO2 recovered to 72%, EtCO2 to 39 mmHg, heart rate settled to 160 bpm, and MAP stabilized at 59 mmHg. No vasopressors were required. The surgical team successfully completed a left hemicolectomy with transversostomy and Hartmann's closure after four hours (Figure 3).

A final 9.6 mL/kg of fresh frozen plasma was transfused to address total operative losses. The final fluid balance was +142 mL, with estimated blood loss of 30 mL and insensible losses of 8 mL/kg/h.

Postoperatively, the patient was transferred back to the neonatal intensive care unit and maintained on invasive mechanical ventilation until the 38th day of life, when he was successfully extubated. His recovery was complicated by nosocomial infections, requiring prolonged courses of broad-spectrum antibiotics. Gastrointestinal management initially involved total parenteral nutrition, with enteral feeding restarted approximately two weeks after the laparotomy. Neurological monitoring through serial transfontanellar ultrasounds revealed normal arterial flow and the absence of intraventricular hemorrhage. On his 56th day of life, the infant was transferred in improved condition to a specialized cardiac center, where he underwent a Rashkind balloon atrial septostomy. At 18 months, he weighs 11 kg and is clinically well (SpO2 86% while crying), with a well-functioning transversostomy; cardiology follow-up continues while definitive correction of d-TGA is awaited.

## Discussion

Emergency management when transfer is not feasible

Management in high-volume pediatric cardiac centers remains the preferred model for patients with complex CHD, but that advantage depends on timely access to specialist care, transport feasibility, and coordinated referral pathways [7]. Unfortunately, the referral model can fail when specialized care is most needed [8,9]. With no viable alternative for transfer, our team was confronted with the conflict between waiting for management in a specialized center and the clinical necessity for emergent intervention. Choosing to proceed locally prioritized immediate physiological stabilization over the risks of awaiting transfer and further aggravating the patient’s condition. Several concrete elements allowed the team to optimize local care under these constraints: anesthetic care was provided throughout by two pediatric anesthesiologists; remote expert consultation with a pediatric cardiac anesthesiologist defined individualized thresholds for intervention; a structured preoperative multidisciplinary briefing was conducted to preassign team roles; vasoactive infusions were precalculated and prepared in advance; external defibrillation pads were placed preemptively, given the high risk of intraoperative arrest; and a deliberate shift to multimodal noninvasive monitoring was made once invasive access failed. Across the entire perioperative course, team coordination and shared mental models were reinforced by structured communication, without delays in decision-making. The remote consultation with a pediatric cardiac anesthesiologist used in this case follows the same underlying logic described by recently reported surgical CHD telementoring platforms, which have shown that synchronous, real-time remote guidance can be delivered to centers without on-site expertise while supporting local decision-making [10]. However, the available literature on pediatric cardiac telemedicine has focused mainly on surgical telementoring, imaging, and perioperative or postoperative teleconsultation rather than on anesthesia-specific teleconsultation, and dedicated evidence for this exact scenario was not identified. In this setting, teleconsultation and structured preoperative preparation appear to have helped maximize safety standards under adverse local constraints.

Cardiac-NEC overlap and intraoperative goals

**Figure 4:**
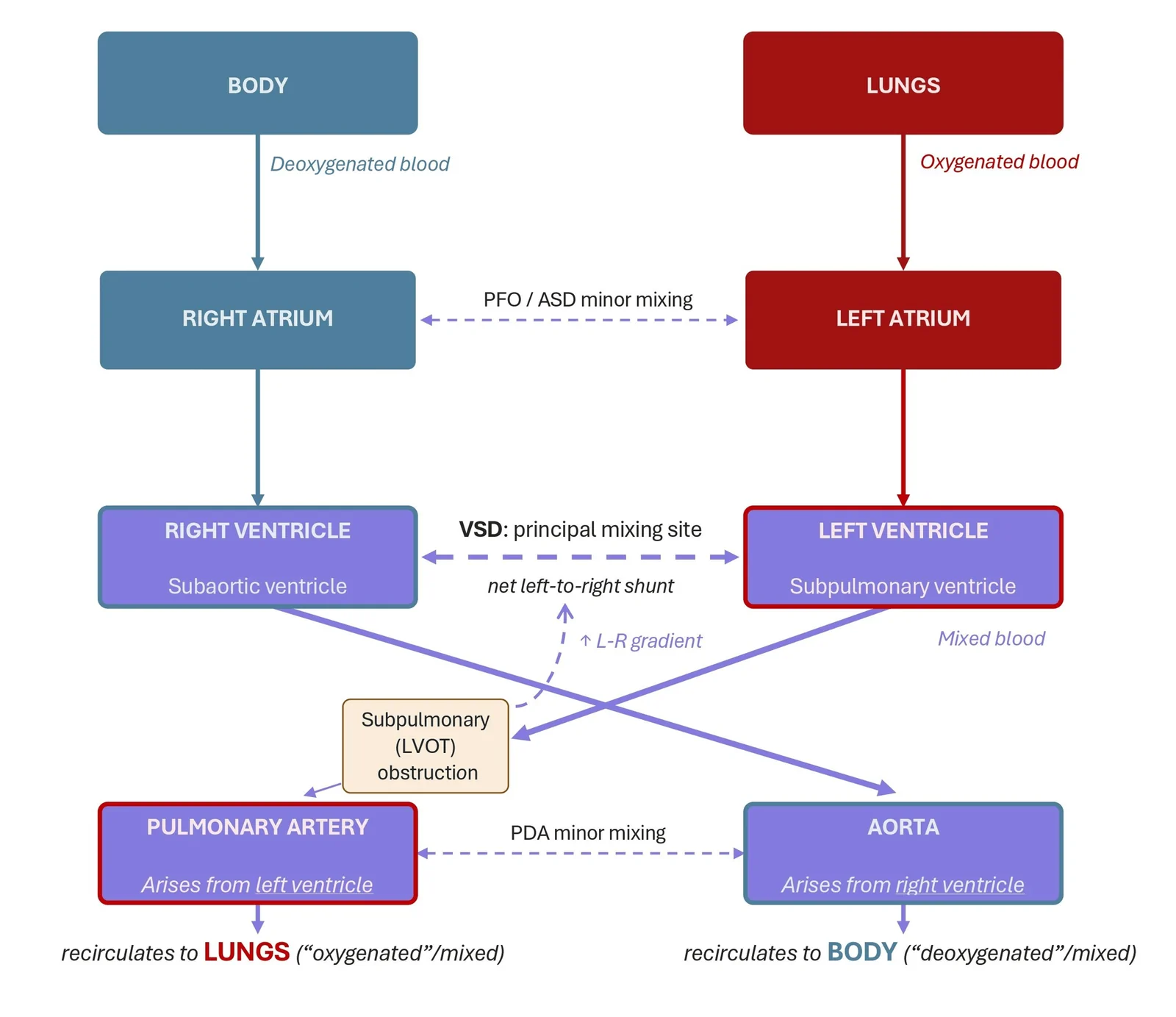
Schematic representation of the parallel circulations and intercardiac mixing sites in dextro–transposition of the great arteries. In the systemic circuit (blue outline), deoxygenated blood returns to the right atrium, crosses the right ventricle (subaortic), and is ejected into the aorta; in the pulmonary circuit (red outline), oxygenated blood returns to the left atrium, crosses the left ventricle (subpulmonary), and is ejected into the pulmonary artery. Diagonal lines illustrate the ventriculoarterial discordance that defines d-TGA: the aorta arises from the right ventricle and the pulmonary artery from the left ventricle. Because these circuits run in parallel, survival depends on mixing (purple fill) at the PFO/ASD, VSD, and PDA; in this patient, the large VSD was the principal mixing site. Severe LVOT obstruction restricted flow into the pulmonary artery, raising left ventricular pressure and augmenting the left-to-right VSD shunt, diverting oxygenated blood into the right ventricle and aorta to support systemic oxygen delivery. ASD, atrial septal defect; L-R, left-to-right; LVOT, left ventricular outflow tract; PDA, patent ductus arteriosus; PFO, patent foramen ovale; VSD, ventricular septal defect.

In patients with d-TGA, the VSD ensures intercirculatory mixing, and the subpulmonary obstruction increases pressure within the subpulmonary ventricle, diverting oxygenated blood across the VSD into the subaortic ventricle and the aorta, thereby promoting a left-to-right shunt that maintains systemic oxygen delivery (Figure 4) [1].

However, lowering systemic vascular resistance (SVR) or subpulmonary ventricular pressure narrows the gradient sustaining this mixing and can precipitate acute systemic desaturation and cyanosis, with pulmonary flow remaining dependent on the PDA [1]. Sepsis associated with NEC may reduce SVR, potentially worsening systemic desaturation by reducing the pressure gradient supporting effective mixing [3]. As cNEC is believed to result from mesenteric circulatory insufficiency, any reduction in SVR compromises the pressure gradient necessary to maintain bowel perfusion, worsening the patient's clinical status.

In infants with CHD, ischemia and hypoxic damage are the dominant risk mechanisms proposed in the pathophysiology of NEC, and the specific risk profile may differ by subtype: cyanotic lesions, such as d-TGA, predispose to a generalized hypoxic state that can itself facilitate NEC development [2,3,5]. There is ongoing discussion about whether initial management should target the CHD to prevent or reverse NEC occurrence; however, once perforation has occurred, the need for emergency surgery prevails over this sequencing discussion [2,3,5]. Thus, our intraoperative goals were maintenance of preload, cardiac output, HR, and SVR and avoidance of myocardial depression in order to prevent further worsening of perioperative systemic arterial desaturation [1,6].

The clinical spectrum of d-TGA is complex and variable, and there is no single anesthetic technique that has been shown to be beneficial [6]. The approach with fentanyl, sevoflurane, and ketamine was preferred due to their synergistic ability to ensure hemodynamic stability and maintain SVR.

The integration of cerebral rSO2, SpO2, and SpHb, the latter functioning as a postductal oximeter, provided a continuous assessment of systemic oxygen delivery. In addition, SpHb allowed for real-time assessment of hemoglobin trends without the need for repetitive invasive sampling. Ideally, continuous invasive arterial blood pressure and an indicator of pulmonary vascular resistance would have been the monitoring standard [6]. However, due to the infant's critical status, prematurity, and the emergency nature of surgery in a nonspecialized center, these invasive measures were unfeasible, requiring reliance on noninvasive trends instead.

The role of NIRS 

NIRS is not yet a routine monitor, but it has been documented in neonatal populations [11,12]. It is a noninvasive device that measures regional tissue oxygenation. Its interpretation allows optimization of various management strategies to address the balance between tissue oxygen delivery and metabolic demand [13]. In conditions of limited global oxygen delivery, as in CHD, optimizing the distribution of cardiac output is a critical goal that can be monitored with this technology.

It is worth noting that during early systemic hypoperfusion, preferential redistribution of cardiac output may help preserve cerebral oxygenation ("brain sparing"), but this compensatory mechanism can become impaired in critically ill neonates [12]. In the context of NEC and prematurity, Kuik et al. observed frequent disturbances in cerebral autoregulation during neonatal laparotomy, suggesting that cerebral oxygenation may also fluctuate substantially during surgery; although observational and not designed to evaluate clinical benefit, these findings point to a potential benefit of continuous cerebral rSO2 monitoring in high-risk neonates [14]. In fact, changes in cerebral rSO2 may precede hemodynamic deterioration, which could position NIRS as a potential early warning system [12,15]. In our patient, the drop in cerebral rSO2 was interpreted as an indicator of a systemic oxygen supply-demand mismatch and not only as a regional event, which prompted therapeutic intervention.

It is also important to consider that absolute rSO2 values in cyanotic CHD can be complex to interpret due to individual compensatory mechanisms such as chronically increased hemoglobin levels [15]. Therefore, various authors emphasize that the trend in values is often more reliable than the absolute number, and it should be weighed alongside clinical examination, blood gases, imaging, and hemodynamic assessment [11,13-15].

Overall, uncertainty remains regarding NIRS and long-term outcomes, as highlighted by a recent systematic review by Hansen et al. This meta-analysis of 25 randomized clinical trials found no significant difference in all-cause mortality, serious adverse events, or persistent cognitive/neurological deficits between groups with access to cerebral NIRS and those without. However, the certainty of evidence was very low because of substantial risk of bias and insufficient sample size [16]. While, in our case, the cerebral NIRS trend supported a multifactorial decision for a proactive transfusion strategy, high-quality evidence is still needed to determine its efficacy and elucidate its role in standard care, particularly in neonates with complex CHD undergoing noncardiac surgery.

Neonatal transport and cardiac surgery centers

Regarding neonatal transport to specialized cardiac centers, it is well established that regionalized cardiac care improves outcomes when transfer is timely and feasible, given that high-volume centers generally have lower mortality and lower costs [7]. Within functioning regionalized networks, transferred neonates with critical CHD can have similar short-term survival rates to those born at the cardiac surgery center [7,17]. However, that benefit depends on the network actually working. Neonatal regionalization remains limited by barriers at patient, hospital, administrative, and policy levels. As noted above, referral models can fail. The same broader transport literature identifies inadequate equipment, low transfer-team skill, lack of centralized transport organization, unclear information exchange, and administratively constrained transport models as major system vulnerabilities [8,9]. This has implications for policy and organization at peripheral centers, including the need for protocols for stabilization, structured communication with the referral cardiac team, thresholds for proceeding locally, and regular multidisciplinary simulation training. These elements should be considered alongside, and not as a substitute for, continued investment in regionalized transport pathways, since most patients with critical CHD still stand to benefit from timely transfer to a specialized center.

Limitations

Only cerebral NIRS was used in this case; a separate and growing body of evidence specifically addresses somatic/splanchnic NIRS as a tool to help diagnose or monitor NEC itself [18]. Combining cerebral and somatic sensors could, in principle, have provided complementary information, both on systemic oxygen delivery (cerebral) and on evolving bowel ischemia (somatic), and represents a reasonable direction for future practice in similar emergencies [18].

As with any single case report, the physiological reasoning presented cannot be generalized into a treatment protocol. The evidence linking the use of cerebral NIRS to improved long-term neurological outcomes remains of low certainty, and the clinical improvement observed in this patient cannot be causally attributed to NIRS monitoring alone [16].

## Conclusions

In conclusion, while specialized pediatric cardiac centers remain the preferred setting for managing neonates with complex CHD, this case demonstrates that life-saving emergency surgery can be successfully performed in nonspecialized facilities when transfer is impossible. Successful management depended on meticulous multidisciplinary planning, remote expert consultation, and physiological goal-directed anesthesia supported by multimodal noninvasive monitoring. The integration of cerebral NIRS and SpHb provided an early warning sign, enabling a proactive transfusion strategy that stabilized systemic oxygen delivery and improved hemodynamic stability, avoiding catecholamine-induced myocardial stress. Ultimately, this report underscores how leveraging teamwork, available monitoring, and physiological understanding can bridge the gap between ideal gold standards and the practical realities of emergency neonatal surgery in centers without specialized pediatric cardiac facilities. It supports a discussion not only of clinical improvisation but also of system resilience, transport limitations, and the need for stronger shared pathways between surgical, neonatal, and cardiac teams.
